# Supplementary material for: Data-driven inference of Boolean networks from transcriptomes to predict cellular differentiation and reprogramming
Source: NPJ Syst Biol Appl. 2025 Sep 26;11:105. doi: 10.1038/s41540-025-00569-z (PMC12475257; doi:10.1038/s41540-025-00569-z)
Supplement: Supplementary file 1 — Supplementary Information [file 41540_2025_569_MOESM1_ESM.pdf]

SUPPLEMENTARY DATA

- Supplementary Data 1. Differential gene expression analysis from scRNA-seq RE1 experiment.
- Supplementary Data 2. Primer sequences, siRNAs, and viral constructs used in the study. The primers, siRNAs, and lentiviral constructs are detailed in the indicated work sheets, respectively.

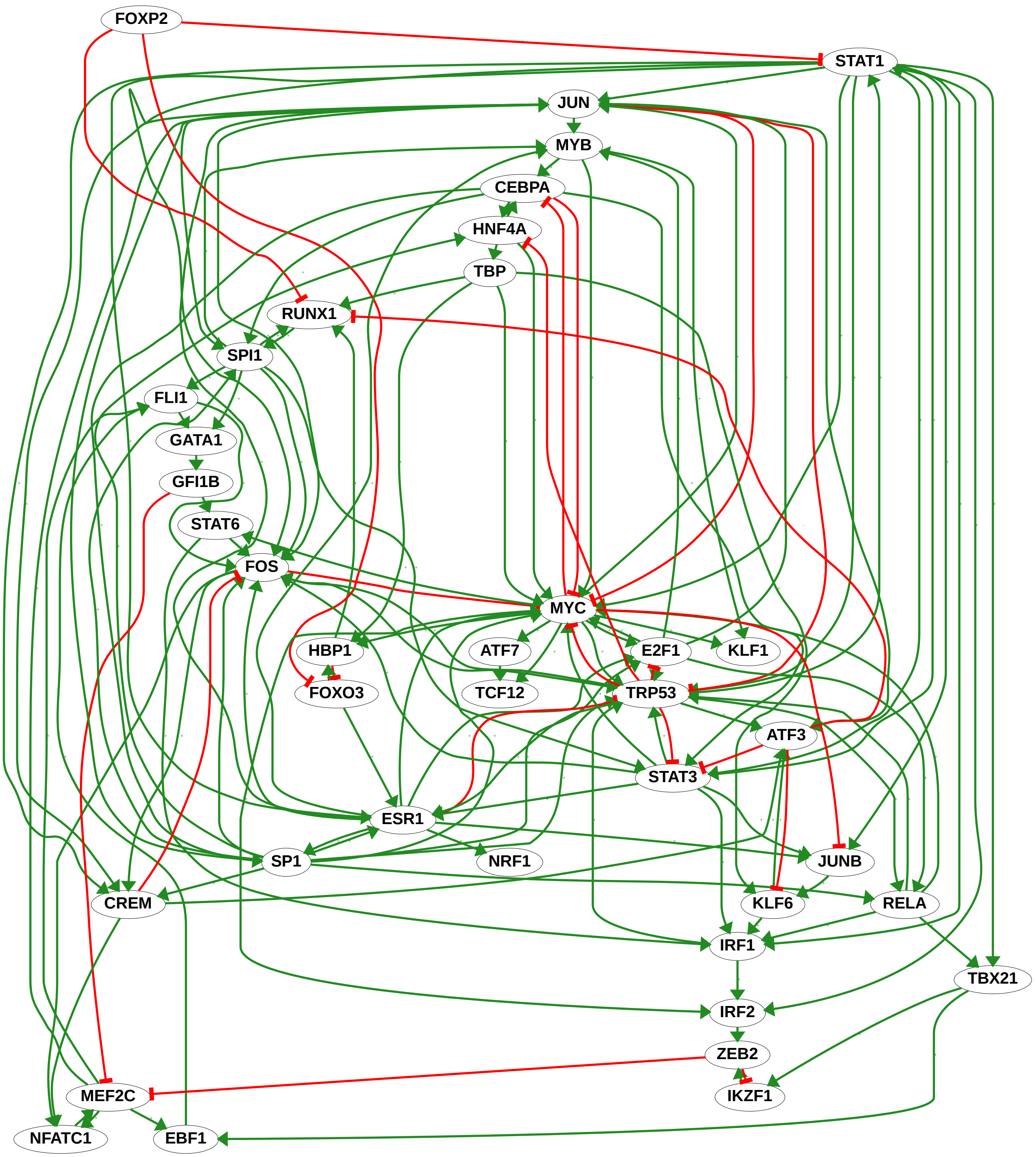

Supplementary Figure 1: Network of 39 components and 137 arcs obtained by component selection using BoNesis.

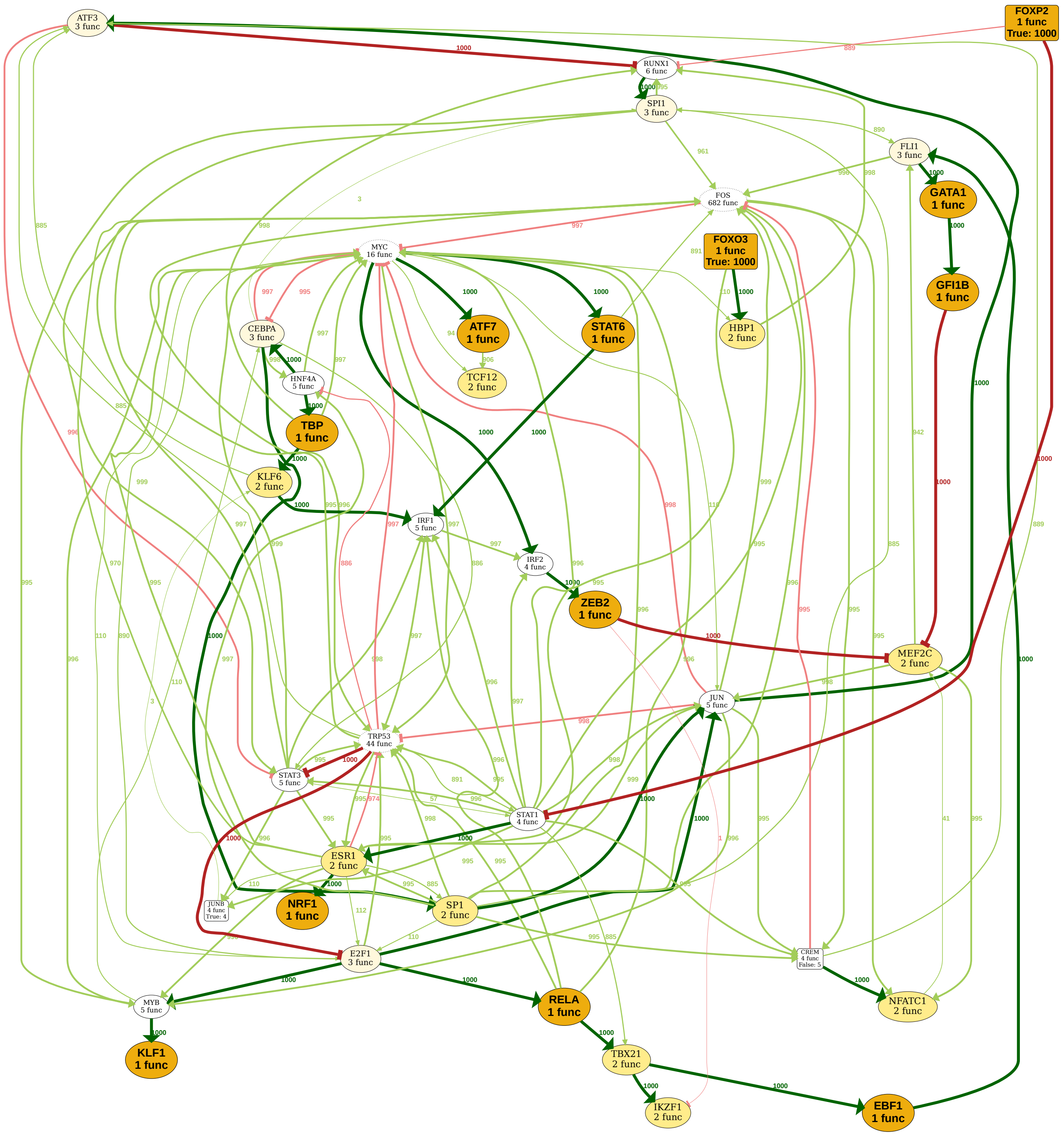

Supplementary Figure 2: Variability analysis Boolean functions in sampled ensemble of models. The label of each node includes the number of different local Boolean functions in the sampled Boolean network set. Orange indicates a unique shared function, yellow only 2 different functions. Edge is labeled with the number of Boolean networks that utilizes the influence (over the 1 000 sampled), with its thickness scaled accordingly.

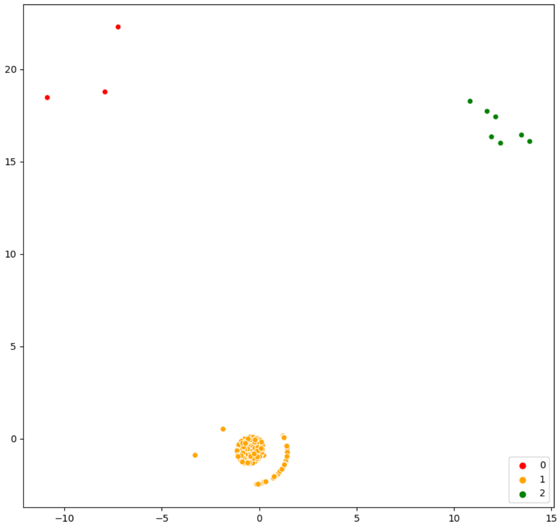

A MDS with 250 models.

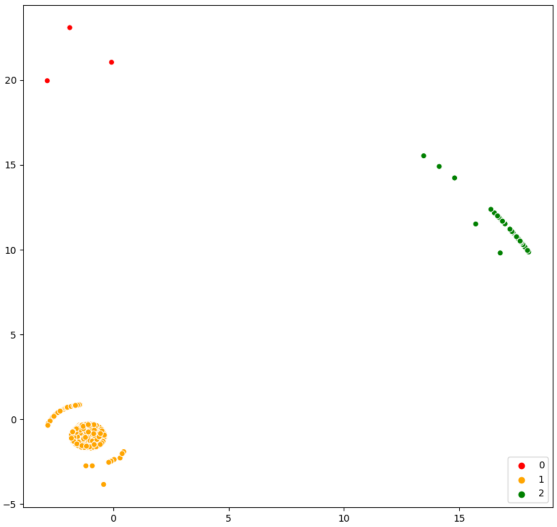

B MDS with 500 models.

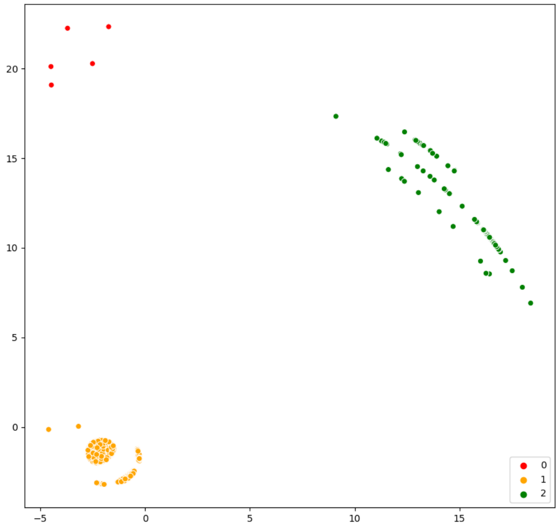

C MDS with 1000 models.

Supplementary Figure 3: MDS highlights 3 groups of models.

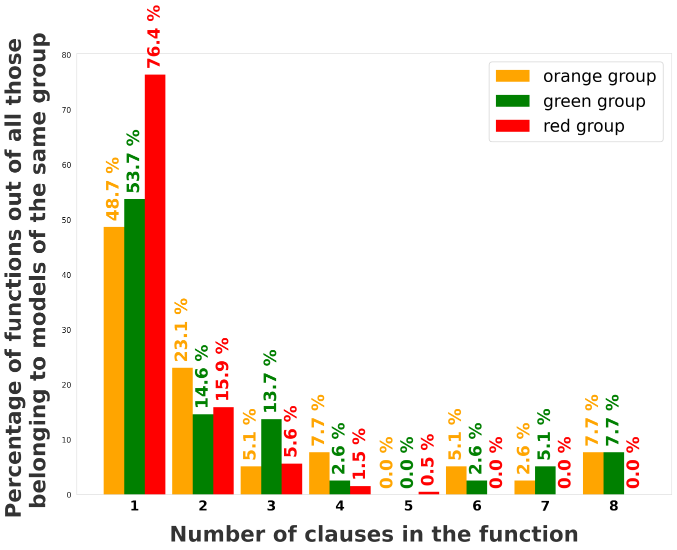

Supplementary Figure 4: Distribution of the functions of the models according to the number of clauses they are made up, by group.

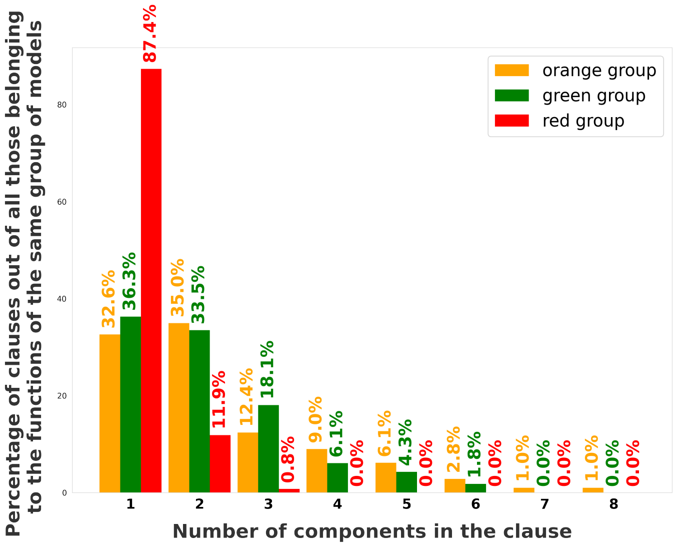

Supplementary Figure 5: Distribution of the clauses of the models according to the number of components they are made up, by group.

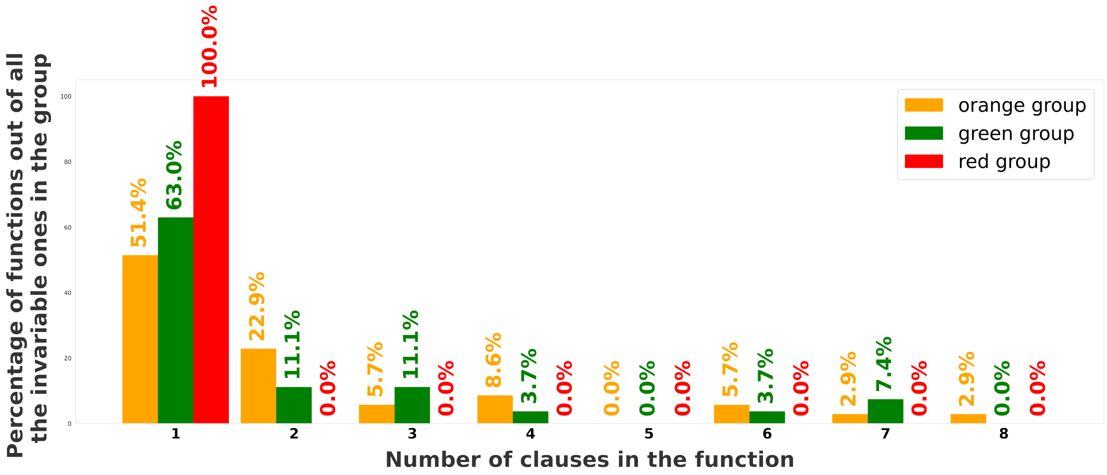

Supplementary Figure 6: Distribution of the invariable functions of the models according to the number of clauses they are made up, by group.

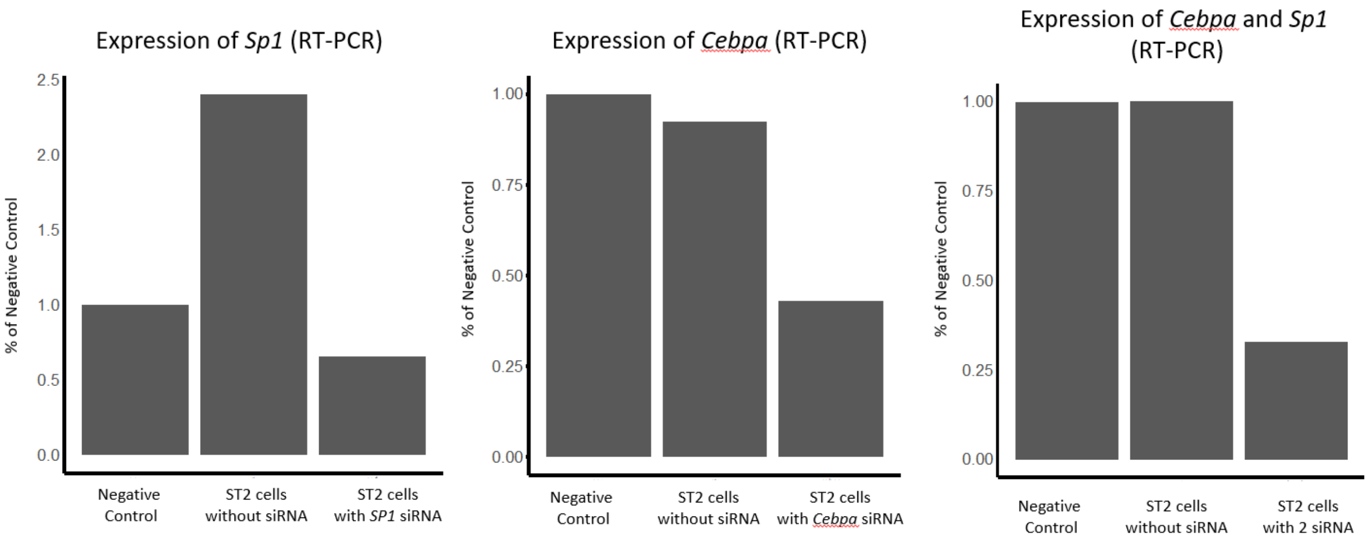

siRNA knockdown efficiency in ST2 (n=1): *Sp1*: 35%; *Cebpa*: 67%, *Cebpa* + *Sp1*: 68%

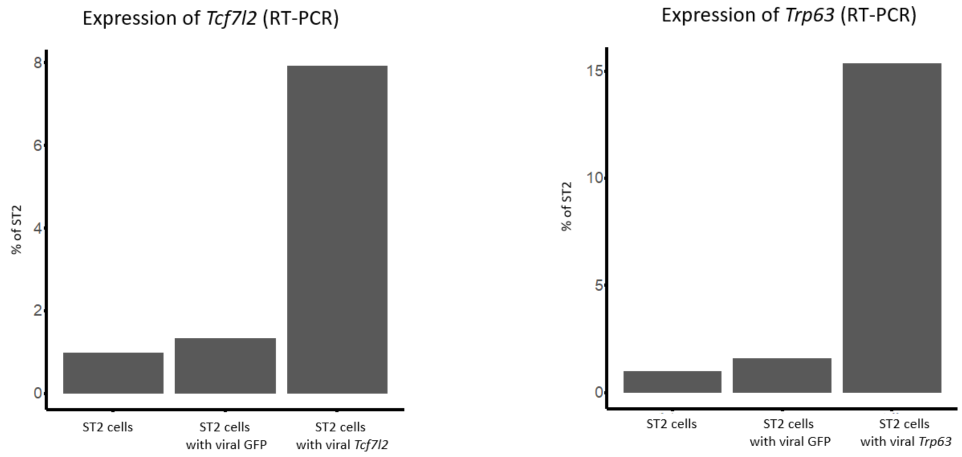

viral knock-in increase expression in ST2 (n=1): *Tcf7l2*: 8 fold; *Trp63*: 15 fold comparing to ST2

Supplementary Figure 7: Experimental validation of case study 2: siRNA and viral transfection test

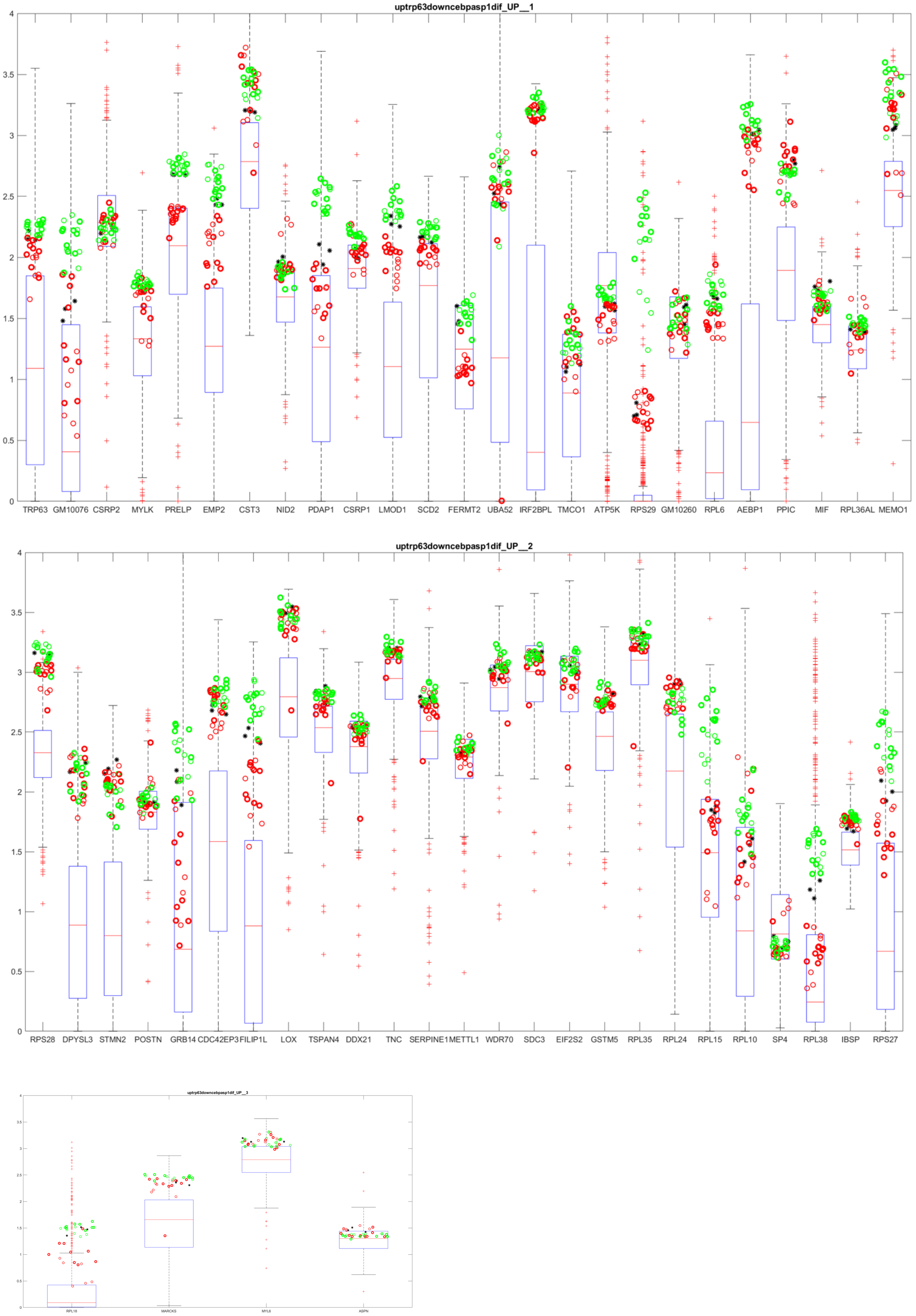

Supplementary Figure 8: Differentially expressed upregulated genes in the RE1 single cell experiment are also upregulated in the bulk RNA-seq data of osteoblasts vs adipocytes. Bulk RNA-seq data (TPM) of osteoblasts (green) and adipocytes (red) are plotted for upregulated sc DEGs (x-axis). Boxplots indicate the gene specific backgrounds from 63 mouse tissues (see methods)

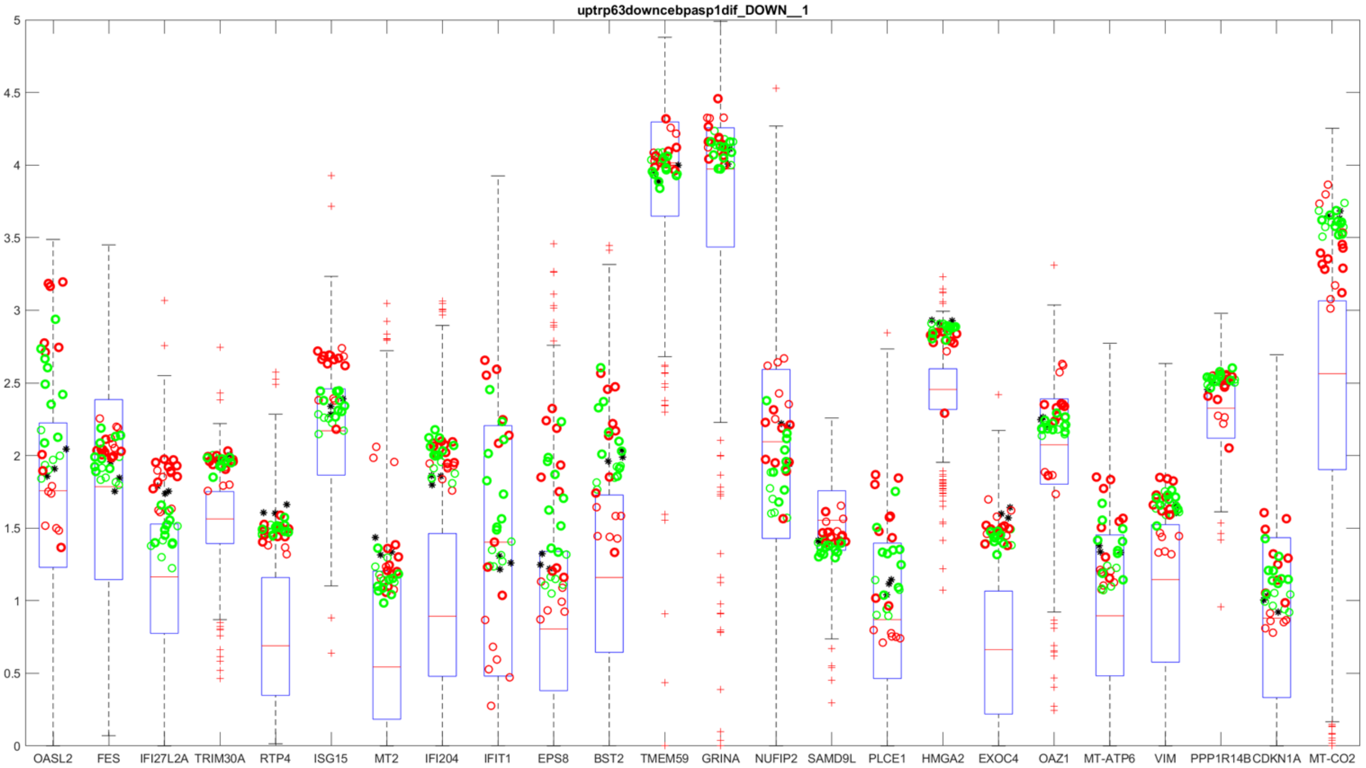

Supplementary Figure 9: Differentially expressed downregulated genes in the RE1 single cell experiment are partially also downregulated in the bulk RNA-seq data of osteoblasts vs adipocytes. Bulk RNA-seq data (TPM) of osteoblasts (green) and adipocytes (red) are plotted for downregulated sc DEGs (x-axis). Boxplots indicate the gene specific backgrounds from 63 mouse tissues (see methods).
